# Food Intake REstriction for Health OUtcome Support and Education (FIREHOUSE) Protocol: A Randomized Clinical Trial

**DOI:** 10.3390/ijerph17186569

**Published:** 2020-09-09

**Authors:** Sophia Kwon, Jessica Riggs, George Crowley, Rachel Lam, Isabel R. Young, Christine Nayar, Maria Sunseri, Mena Mikhail, Dean Ostrofsky, Arul Veerappan, Rachel Zeig-Owens, Theresa Schwartz, Hilary Colbeth, Mengling Liu, Mary Lou Pompeii, David St-Jules, David J. Prezant, Mary Ann Sevick, Anna Nolan

**Affiliations:** 1 Department of Medicine, Division of Pulmonary, Critical Care and Sleep Medicine, School of Medicine, New York University, New York, NY 10016, USA; Sophia.Kwon@nyulangone.org (S.K.); jarigg@gmail.com (J.R.); George.crowley@nyumc.org (G.C.); Rachel.lam@nyumc.org (R.L.); Isabel.Young@nyumc.org (I.R.Y.); Christine.Nayar@nyumc.org (C.N.); Maria.Sunseri@nyumc.org (M.S.); Mena.mikhail@nyumc.org (M.M.); Dean.Ostrofsky@nyumc.org (D.O.); Arul.Veerappan@nyumc.org (A.V.); 2 Bureau of Health Services and Office of Medical Affairs, Fire Department of New York, Brooklyn, NY 11201, USA; Rachel.Zeig-Owens@fdny.nyc.gov (R.Z.-O.); Theresa.Schwartz@fdny.nyc.gov (T.S.); Hilary.Colbeth@fdny.nyc.gov (H.C.); David.Prezant@fdny.nyc.gov (D.J.P.); 3 Pulmonary Medicine Division, Department of Medicine, Montefiore Medical Center and Albert Einstein College of Medicine, Bronx, NY 10461, USA; 4 Department of Population Health, Division of Biostatistics, New York University School of Medicine, New York, NY 10016, USA; Mengling.liu@nyumc.org; 5 Department of Environmental Medicine, School of Medicine, New York University, New York, NY 10016, USA; 6 Department of Population Health, Division of Health and Behavior, Center for Healthful Behavior Change, School of Medicine, New York University, New York, NY 10016, USA; MaryLou.Pompeii@nyulangone.org (M.L.P.); stjules@unr.edu (D.S.-J.); Mary.Sevick@nyulangone.org (M.A.S.); 7 Departments of Medicine, Division of Endocrinology, School of Medicine, New York University, New York, NY 10016, USA

**Keywords:** metabolic syndrome, biomarkers, lung injury, World Trade Center, particulate matter, 9/11, metabolomics, firefighters, Mediterranean diet

## Abstract

Fire Department of New York (FDNY) rescue and recovery workers exposed to World Trade Center (WTC) particulates suffered loss of forced expiratory volume in 1 s (FEV_1_). Metabolic Syndrome increased the risk of developing WTC-lung injury (WTC-LI)_._ We aim to attenuate the deleterious effects of WTC exposure through a dietary intervention targeting these clinically relevant disease modifiers. We hypothesize that a calorie-restricted Mediterranean dietary intervention will improve metabolic risk, subclinical indicators of cardiopulmonary disease, quality of life, and lung function in firefighters with WTC-LI. To assess our hypothesis, we developed the Food Intake REstriction for Health OUtcome Support and Education (FIREHOUSE), a randomized controlled clinical trial (RCT). Male firefighters with WTC-LI and a BMI > 27 kg/m^2^ will be included. We will randomize subjects (1:1) to either: (1) Low Calorie Mediterranean (LoCalMed)—an integrative multifactorial, technology-supported approach focused on behavioral modification, nutritional education that will include a self-monitored diet with feedback, physical activity recommendations, and social cognitive theory-based group counseling sessions; or (2) Usual Care. Outcomes include reduction in body mass index (BMI) (primary), improvement in FEV_1_, fractional exhaled nitric oxide, pulse wave velocity, lipid profiles, targeted metabolic/clinical biomarkers, and quality of life measures (secondary). By implementing a technology-supported LoCalMed diet our FIREHOUSE RCT may help further the treatment of WTC associated pulmonary disease.

## 1. Introduction

Metabolic Syndrome (MetSyn) is defined as having at least three of five risk factors (abdominal obesity, insulin resistance, hyperglycemia, dyslipidemia, and hypertension) that increase the likelihood of cardiovascular disease [1]. MetSyn afflicts more than 30 percent of adults (>47 million Americans) and is associated with impaired lung function [2,3]. Our group has focused on defining the development of World Trade Center-particulate matter (WTC-PM) associated lung disease in the context of MetSyn in the well-phenotyped Fire Department of New York (FDNY) Cohort [2,3,4,5,6,7,8,9,10,11].

Nearly half of chronic obstructive pulmonary disease (COPD) patients demonstrate the presence of MetSyn phenotypic characteristics [12,13,14]. Systemic inflammation, which is often seen in MetSyn, is implicated in lung function loss, the development of chronic obstructive airways dysfunction (OAD), vascular disease, and cancer [15,16,17,18,19]. Additionally, longitudinal investigations indicate that lower baseline forced expiratory volume in one second (FEV_1_) was an independent predictor of MetSyn [10,11]. Our recent investigations indicate that MetSyn components, most strongly dyslipidemia and abdominal obesity, are significant predictors of World Trade Center-lung injury (WTC-LI), as defined by FEV_1_ less than the lower limit of normal (LLN).

Although one major contributor to poor lung mechanics is from mechanical load-induced stress secondary to abdominal obesity, our prior work shows near equivalent contribution from dyslipidemia in those with WTC-LI [2,10,11]. Inflammatory profiles from serum samples collected within three months of 9/11 showed that dyslipidemia predicted WTC-LI even after adjusting for body mass index (BMI), and was in fact a stronger predictor than obesity [2,3,4,5,6,7,8,9,10,11,15,20,21]. In light of these findings, we focused our work on the inflammatory effects of lipids in the development of particulate matter (PM)-induced lung injury [22]

To investigate the potential reversibility of WTC-LI by direct impact on MetSyn risk factors, we focus on calorie-restricted (CR) Mediterranean diets based on recent studies showing their ability to attenuate lipid levels [20,23,24]. Modifying MetSyn through diet and exercise has been used successfully in other studies to directly impact lung disease. Dietary interventions in OAD patients improved FEV_1_ and forced vital capacity (FVC) by as much as 22 percent in as little as 15 days [25,26]. Using a low calorie diet, investigators achieved a 20 kg loss over a 6-month period, and found that every 10 percent relative loss of weight led to significant improvement of FVC by 92 mL and FEV_1_ by 73 mL [27]. It has been documented that patients with decreased BMI from 37 to 32 kg/m^2^ also had increased FEV1 and FVC [28].

Technology-supported behavioral interventions have shown enhanced data collection and improved adherence to diet plans [29,30]. Mobile technology allows clinicians to deliver targeted behavioral interventions and deliver counseling remotely, increasing scalability. We have previously developed an innovative weight loss intervention involving: (1) cloud-based self-monitoring of behavior (diet and physical activity) with automated feedback; (2) theory-based behavioral counseling delivered via WebEx (Cisco WebEx, Milpitas, CA, USA) group sessions; (3) standardized education to minimize calorie intake and enhance energy expenditure [29].

In the context of our prior findings, as well as the previously discussed literature linking nutritional modification to health benefits, we have chosen to explore the therapeutic potential of ameliorating MetSyn phenotype in our WTC-exposed cohort [2,3,4,5,6,7,8,9,10,11,21]. In the current study, we are implementing a technology-supported, low calorie Mediterranean (LoCalMed) dietary intervention to improve metabolic risk factors in subjects with WTC-LI. We hypothesize that our dietary intervention will have a therapeutic effect on the clinical endpoints of body mass index (BMI), FEV_1_, fractional nitric oxide (NO) concentration in exhaled breath (FeNO), pulse wave velocity (PWV), lipid profiles, targeted biomarkers and quality of life measures.

## 2. Methods

### 2.1. Design and Setting of the Study

FIREHOUSE is a 6-month randomized, controlled, two-arm, parallel, un-blinded, exploratory clinical trial, with 1:1 allocation, in overweight (specifically for this study our inclusion criteria was a BMI > 27 kg/m^2^) FDNY firefighters with documented WTC-LI (defined as FEV_1_ < LLN post exposure). While overweight is defined as having a BMI of 25.0 to <30.0 kg/m^2^, and subjects are considered obese if there BMI is ≥30.0 kg/m^2^ [31], for the purposes of our study we wanted to enroll subjects that were overweight with a BMI that was greater than 27 kg/m^2^. This was by design since our power analysis and primary outcome assessment was targeting at least 1–2 units of BMI change in the intervention group [32,33,34,35].

Ethics: The trial and all proposed analysis will be performed in accordance with the New York University (NYU)-Institutional Review Board, Protocol 17-00127.

#### 2.1.1. Participant Characteristics

**Figure 1:**
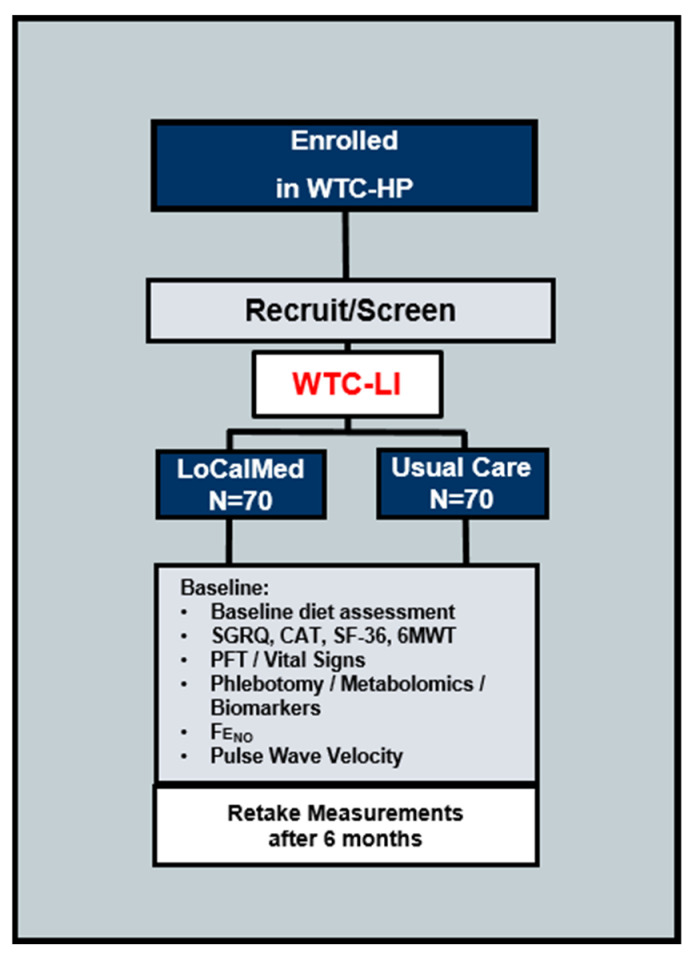
Schematic of the Food Intake REstriction for Health OUtcome Support and Education (FIREHOUSE) trial subject selection, illustrating study cohort World Trade Center-lung injury (WTC-LI) as a subset of the larger World Trade Center-Health Program (WTC-HP) enrolled Fire Department of New York (FDNY) first responders. WTC-HP, World Trade Center health program; SGRQ, St. George’s Respiratory Questionnaire; CAT, Computerized Axial Tomography; SF-36, 36-Item Short Form Survey Instrument; 6MWT, Six Minute Walk Test; FeNO, fractional nitric oxide (NO) concentration in exhaled breath.

Inclusion and exclusion criteria for study enrollment detailed in Table 1. In brief, participants are eligible for inclusion if they are FDNY firefighters with documented WTC exposure, enrolled in the FDNY WTC-health program (WTC-HP), and have WTC-LI as defined by an FEV_1_ < LLN after exposure, Table 1 and Figure 1. This FIREHOUSE trial will be performed in accordance with the Declaration of Helsinki and Good Clinical Practice and the protocol is approved by the New York University IRB #17-00127.

Participant recruitment will include: (1) direct mailings; (2) telephone contact; (3) email; (4) self-referral. Recruitment efforts will be supported by a study website that provides general information about the study and answers frequently asked questions. The website will also facilitate study activities during the intervention period.

**Figure 2:**
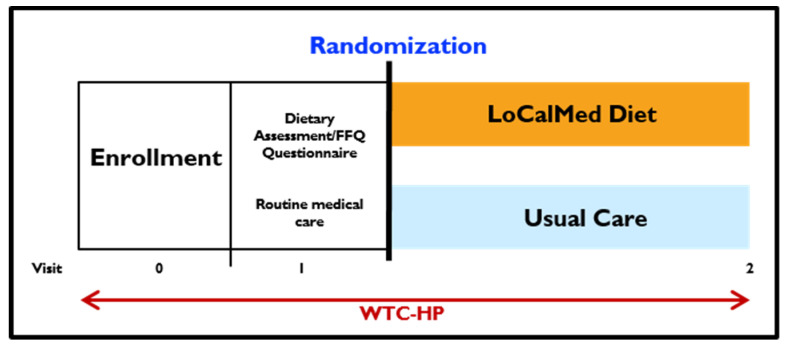
Study design of FIREHOUSE Trial. All participants will be enrolled during the enrollment period at Visit 0. At Visit 1, cohort will complete the dietary assessment/food frequency questionnaire, receive routine medical care, and obtain baseline measurements. Randomization will then occur and groups will be assigned to either LoCalMed or Usual Care groups. LoCalMed Dietary and Intervention group will receive weekly or biweekly technology-driven nutrition visits as scheduled, whereas Usual Care group will receive quarterly flyers to perform routine medical care. Follow-up measurements at Visit 2 will be collected at the end of the 6-month intervention for both groups. The World Trade Center-Health Program (WTC-HP) will be available throughout the time period to all participants to provide other medical care.

Participants will be randomized to either Usual Care (control arm) or LoCalMed diet plus technology-assisted nutrition education and behavior modification (intervention arm), Figure 2. Each study arm is active for six months following enrollment. Participants will return after six months for a repeat of all measures. All participants will be informed of pertinent clinical assessment conclusions and offered all of the intervention materials at the study’s conclusion, Table 2 and Table S1.

#### 2.1.2. FIREHOUSE Intervention Usual Care Group

The Usual Care group serves as a control (reference) arm, with target enrollment of *N* = 70. In addition to standard care from their WTC-HP primary provider, participants in the Usual Care group will receive newsletters every five weeks during the intervention from study staff related to: (1) mindfulness; (2) sleep; (3) staying healthy year-round; (4) disease self-management. As an added incentive for remaining in the study, Usual Care participants will be provided access to educational and behavioral materials upon completion of their 6-month measurement visit. The LoCalMed group will be enrolled in a 6-month, technology-supported, educational-behavioral program designed to promote a calorie-restricted, Mediterranean-style diet and regular, moderate-intensity physical activity. At baseline, participants will be provided in-person, group-based technology training, and will receive handouts with the following study goals: (1) weight loss of ≥seven percent at 6-months; (2) saturated fat intake ≤seven percent of kcal; (3) ≥150-min per week of moderate-intensity physical activity. After the initial technology training session, all intervention-related activities will be delivered remotely via smartphones. The LoCalMed program will consist of two main parts: (1) mobile self-monitoring with feedback; (2) remote group-based education and behavioral counseling.

##### Mobile Self-Monitoring

Participants will be provided a 6-month subscription for the commercial diet diary application MyNetDiary (MND, MyNetDiary, Inc., Marlton, NJ, USA) and directed to record their body weight weekly, all food and drink consumption, and any physical activity as it occurs. If needed, participants are provided with a digital bathroom scale for monitoring body weight during the intervention.

Self-monitoring of health behaviors (e.g., diet) and outcomes (e.g., body weight) is thought to promote behavioral change in part by bringing these factors to individuals’ conscious attention, thereby enhancing vigilance. In addition to providing a digital platform for self-monitoring, MND generates real-time feedback on nutrient intakes across foods, meals, and meal-days, as well as daily and weekly summaries of recorded physical activities. The multi-level organization of data in MND assists with information processing, which may reduce information burden that can lead to oversimplified heuristics (aka “food rules”) and burnout. To further limit information burden, MND nutrient output will be restricted to track only calories and macronutrients (carbohydrates, protein, and total, saturated, monounsaturated, and polyunsaturated fats). MND summary reports are updated with new entries, they will permit ecological momentary assessment (EMA) of behaviors at the time, which is thought to augment the effectiveness of self-monitoring.

Adding to the automated feedback provided by MND, the study dietitian will review the participants’ MND accounts weekly for the first four weeks and every second week thereafter during the intervention, and they will provide feedback reports via the participant’s personal (or a study-provided) email account. Reports will contain three parts: (1) adherence to self-monitoring; (2) body weight; (3) saturated fat intake. Feedback on saturated fat is graded as green (≤seven percent of kcal), yellow (>7–10 percent of kcal) or red (>10 percent of kcal), and it includes tailored advice on restricting saturated fat based on participants’ MND records. Within the program, reported body weights are plotted on a personalized weight loss trajectory line graph that is modeled with 95 percent confidence intervals to achieve a seven percent weight loss over six months [36]. In our experience, participants are motivated by the additional accountability that is provided by external monitoring of health behaviors and outcomes by study staff.

##### Education and Behavioral Counseling

Participants will receive group-based education and behavioral counseling (~1-h) each week for the first four weeks, and every other week thereafter during the intervention via the videoconferencing application, WebEx (Cisco WebEx, Milpitas, CA, USA). Group sessions are facilitated by the study dietitian, and anchored by brief animated videos (Powtoon, London, UK), Table 3. After each session, videos will be posted on the study website, which participants can access via a password-protected Brainshark link (Brainshark, Inc., Waltham MA, USA). Brainshark is a video viewing platform that records time spent watching videos and will help assess intervention dose.

Education videos in Table 3 were designed to provide intervention subjects materials for following a calorie-restricted, Mediterranean-style diet, and engaging in regular moderate-intensity physical activity, Table 3. The Mediterranean diet was selected as the dietary pattern for this intervention because of the emphasis on healthy fats. Previous metabolomics analysis of FDNY 9/11 first responders with and without WTC-LI suggested that metabolic pathways affected by dietary fats were risk factors for the condition [22]. This dietary pattern, which promotes whole fruits and vegetables, whole grains, nuts, legumes, fish, healthy oils (namely olive oil), and a limited amount of lean meat and low-fat dairy products, is largely consistent with dietary patterns that are commonly used in weight loss. To assist participants in restricting energy intake, additional education is provided on portion control, meals away from home, meal skipping, and avoiding empty calories (e.g., sugar-sweetened beverages), Table 3.

Behavioral counseling and videos are based on social cognitive theory (SCT), which focuses on the roles of self-referent thought and self-efficacy (i.e., confidence) in adopting and maintaining healthy behaviors. According to SCT, self-efficacy can be derived from four factors: (1) mastery experiences: goal-setting, prior successes, and problem-solving; (2) social modeling: learning by observing others; (3) verbal persuasion: encouragement from others; (4) physiological benefits: improvements in health outcomes (e.g., weight loss), Table 3.

Primary Outcome Assessment is a seven percent decrease in total body weight with a subsequent drop in BMI of roughly 1–2 kg/m^2^ depending on the individual’s height.

Secondary outcome measures include a 7–10 cc increase in FEV_1_ after trial intervention compared to control subjects, as well as an increased lean body mass relative to fat percentage on body-impedance analysis (BIA), reduction in PWV and FeNO, and improved perceived health status as measured by the Short Form 36 (SF-36) and the St. George’s Respiratory Questionnaire (SGRQ), which are both validated quality of life assessment tools available for public use. Details of the primary and secondary endpoints of the FIREHOUSE trial are shown in Table 4.

Additionally, we will collect clinical measurements such as routine vital signs, electrocardiograms (ECGs), routine chemistries, complete blood counts (including white blood cell differentials), liver function tests, and blood lipid profiles. Furthermore, we will collect saliva and stool samples for genomic (ORAgene-Discover; OGR-500; DNAgenotek) and microbiomic analysis (OMNIgeneGut; OMR-200 DNAgenotek), respectively.

### 2.2. Statistical Analysis

Primary statistical modeling will be performed based on the change in outcomes from baseline to 6-months post-intervention between the two randomized groups. Descriptive analysis of all data collected, using typical graphic and numerical exploratory data techniques, is planned. An intention to treat approach will be used to avoid bias from noncompliance and missing outcomes for primary analyses of the treatment effect between two groups. Regression models will be used for modeling treatment effects while adjusting other covariates. Post hoc multiple comparisons tests are applied to adjust for pairwise comparisons.

#### Sample Size and Interim Analysis

The primary objective is to compare the change in BMI in the two groups from pre-intervention to post-intervention after six months (intervention completion). The analysis for this primary objective will be based on the multivariate linear regression with adjustment for potential confounders. The difference for the change of BMI between the two groups will be estimated, and a corresponding 95 percent confidence interval will be calculated.

The sample size of the FIREHOUSE trial was estimated by the power analysis for the primary outcome of the change in BMI predicted between the start and end of the intervention, which was based on a two-sample test to compare the outcome between two groups under 1:1 randomization. According to the preliminary data, we assumed SD = 2 kg/m^2^ and the means of two groups “Usual Care” and “LoCalMed” to be 0 and −1 kg/m^2^, respectively. Furthermore, 6-month interventions with similar calorie targets and behavioral modification with exercise produce a 7–10 percent weight loss [37,38]. A total sample of 128 subjects (64 subjects in each arm) can achieve 80 percent power at α = 0.05. We will recruit 140 subjects accounting for potential loss of around 20 percent drop out.

The interim analysis plan will incorporate two interim looks and one final look, with all looks named as formal interim analysis, Table 5. This study will not be monitored for futility. The first look will be conducted at the completion of 6-month intervention or observation for 30 subjects in each group. This look will use 2-sided α < 0.001. The second look will be one formal interim look, which will be conducted at the completion of 50 subjects in each group with a 2-sided α = 0.007. The final analysis will take place at completion of all subjects in each group, with an O’Brien Fleming boundary for a 3-look design with 2-sided α = 0.033, Table 5 and Figure S1.

### 2.3. COVID-19 Pandemic Precautions

Coronavirus disease 2019 (COVID-19), caused by the severe acute respiratory syndrome coronavirus 2 (SARS-CoV-2), emerged in Wuhan, China, in late 2019 and has since developed into a pandemic. During the FIREHOUSE RCT’s enrollment period, New York State emerged as an epicenter of the global health emergency [39,40,41,42,43,44,45,46,47,48,49].

In response to this novel respiratory pathogen, NYU paused face-to-face clinical research for many studies in order to assess, address and minimize any potential COVID-19-related risks. This impacted our in-person baseline visits as well as final study visits. Meetings with our nutritionist continued for subjects already enrolled since these were designed to be on a video platform.

Once safe to resume these activities, we implemented a COVID-19 pre-visit screening with subjects. This “Research Participant Information Sheet: COVID-19 Updates” informs both newly screened/enrolled subjects as well as subjects returning for their follow-up evaluation about the safeguards that have been put into place to protect against the risk of COVID-19 transmission while restarting certain in-person research activities.

Specifically, subjects were informed that since some study visits need to be conducted in person, participation in these study activities may increase their risk of exposure to the COVID-19 virus. We also discussed that NYU, as with nearly all public venues, has implemented a number of safety policies to protect subjects and to reduce exposure risk. These policies include, among other requirements, screening all persons involved in research visits (staff and study subjects), use of Personal Protective Equipment (PPE), following Social Distancing guidelines when possible, and cleaning and sanitizing rooms and equipment. These protective measures are similar to those most doctors’ offices have implemented in response to COVID-19.

This participant information document also informs subjects that their participation is completely voluntary. Additionally, subjects are provided with the Principal Investigator (PI)’s contact information and given the opportunity to ask any questions that may arise. A copy of this information sheet is also provided to the patient.

Due to the pause, the final study visit timeframe, to the extent to which it could be performed, was extended for at least three additional months. Since spirometry and FeNO assessment may lead to aerosolization, we also suspended their assessment for the purposes of research during the COVID-19 pandemic.

## 3. Discussion

The impact on quality of life and financial burden of WTC-LI are public health concerns. Despite treatment, new cases continue to be identified, and those affected continue to experience morbidity. Our work fits into broader literature that explores the association of poor metabolic health and OAD in those exposed to pollution. Although the mechanisms that lead to lung disease in the context of pollution and metabolic syndrome remain poorly understood, we have shown that modifiable risks such as BMI and dyslipidemia can predict development of WTC-LI. In the FIREHOUSE RCT, we will investigate dietary intervention as a treatment of WTC-LI.

Our prior investigations, from which the FIREHOUSE RCT stems, allowed us to discover and validate a metabolomic signature of WTC-LI, while investigating the effects of confounders found in the entire FDNY cohort such as smoking, obesity, and diet. Since dietary habits have a direct relationship to MetSyn and the metabolome, we propose to alter the subjects’ nutritional status using a Mediterranean style diet.

Our current WTC portfolio lacks insight into dietary patterns and quality of life measures as they pertain to metabolic risk. Furthermore, it lacks direct vascular injury measures and assessments of FeNO and PWV. In the context of our preliminary findings, and due to a clinical need, we propose an RCT to assess the effect of dietary modification on BMI, lung function, vascular injury, and quality of life measures. We measure PWV, a marker of central aortic stiffness, which has implications in cardiovascular disease [50,51,52,53,54]. A meta-analysis of 20 studies showed that modest weight loss (eight percent of the initial body weight) caused a reduction in PWV measured in all arterial segments [55,56]. A systematic review and meta-analysis of RCTs looking at the effect of dietary interventions found that polyunsaturated fatty acids (PUFA) supplementation improved PWV [57,58]. Pulmonary vascular injury occurs early in obstruction, prior to the development of abnormal FEV_1_ [59,60]. We have published that enlarged pulmonary artery/aorta ratio by CT scan and dyslipidemia are biomarkers of WTC-LI [61]. FeNO, a clinically accepted, indirect biomarker of lung disease activity, will be a valuable measure in our population. FeNO is associated with airway hyperreactivity, and several studies show that FeNO increases during obstructive exacerbations [62].

We propose to sample, bank, and measure serum and plasma for biomarker assessment before and after the dietary intervention. Biomarkers are powerful predictors of disease that are useful in defining the efficacy of our intervention. This biorepository will not only allow us to complete and evaluate our current clinical trial but will be valuable to future epigenetic studies of WTC disease. Assaying biologically relevant biomarkers allows for the identification of relevant pathways involved in WTC-LI and helps guide therapies targeting these mediators. We will also obtain peripheral blood mononuclear cells from collected blood that will allow us to assess the genomic and microbiomic signatures pre/post intervention.

There are limitations to this study, and a few will be discussed in this protocol paper. First, the study population is male and largely Caucasian, which may limit generalizability. To limit bias in this un-blinded study, we will randomize after visit one. However, there is a chance that the main research team (not including the nutritionist and technology support providers) will be privy to subject identities and experimenter bias. Given the tightly knit nature of the FDNY community, we also surmise that there could be some sharing of information regarding the protocol between participants in opposing treatment arms. Furthermore, as a behavioral and educational intervention, there is marked variability among individuals with regard to technologic savviness, educational background, willingness to alter diet and exercise, social support, economic means for a healthier diet, and physical ability to exercise. This variability will be equally prevalent in both groups. Similarly, subjects unwilling to participate fully will not pass the inclusion criteria.

The FDNY cohort is highly motivated to improve their disease burden and quality of life. Rescue workers receive routine screening and have an excellent recall of at least 90 percent in prior clinical studies. In addition, there is a strong commitment at the FDNY for this proposed collaborative NYU/FDNY effort to improve the health of these brave individuals. Many participants are aware of our previous findings and have asked for ways to modify their lifestyle with hopes of changing disease outcomes. Hence, it is logical to provide this much-affected cohort with interventions that they may benefit from. This intervention, once validated, may yield meaningful findings for populations exposed to ambient and other occupational particulates.

## Figures and Tables

**Table 1:** Inclusion and exclusion criteria for the randomized controlled clinical trial (RCT) FIREHOUSE Study.

Inclusion Criteria	Exclusion Criteria
Male sex, over age 21 years at enrollment	Pre-existing conditions including (and not necessarily limited to) active cancer, severe heart disease, significant cognitive impairment, eating disorders, significant psychiatric illness, end-stage COPD, severe pulmonary HTN, or organ transplant
FDNY rescue and recovery worker	Concomitant use of interfering medication(s) or devices within one month prior to enrollment
Documented WTC exposure	Severe GI illness that would prevent diet adherence
Enrolled in the FDNY WTC health program	Severe kidney disease requiring dialysis
Willing and able to consent for themselves to study enrollment	Severe liver disease requiring frequent medical intervention
Willing and able to participate in study procedures, to modify their diet and activity level	Participation in other diet modification studies
Able to perform ADLs independently	High-dose steroid (>20 mg prednisone or equivalent) or other hormonal treatments or chemotherapy within one month
Light duty or retired FDNY firefighters	Life expectancy < 6-months
FEV_1_ less than LLN of predicted at any time post 9/11	Recent significant weight loss > five percent TBW within one month
Spirometry within the last 36- months, and at post-9/11 visit	Significant alcohol use
BMI > 27 kg/m^2^ and < 50 kg/m^2^	
Able to demonstrate minimal proficiency using a smart phone	
Have means to accommodate transportation to/from in-person visits	

ADL, activities of daily living; HTN, hypertension; FDNY, Fire Department of New York; WTC, World Trade Center GI, gastrointestinal; TBW, total body weight; FEV_1_, forced expiratory volume in 1 s; LLN, lower limit of normal; BMI, body mass index; COPD, chronic obstructive pulmonary disease. Table 1 includes descriptions of both the inclusion and exclusion criteria that we will use to qualify potential study participants.

**Table 2:** Schedule of enrollment, intervention, and assessment.

	Enrollment	Pre-Randomization Baseline	Post-Randomization		Close-Out
TIMEPOINT (Visit)	0	1	T/N	2	F/U
ENROLLMENT					
Eligibility screen	x				
Informed consent	x				
INTERVENTIONS					
LoCalMed			x	x	
Usual Care				x	
ASSESSMENTS					
Physical exam		x		x	
Phlebotomy		x		x	
EKG/PWV					
FeNO		x		x	
Spirometry		x		x	
Genome		x		x	
Microbiome					
Questionnaires		x		x	x
INSTRUCTIVE COMPONENTS					
Technology training			x	x	
Nutrition consultation			x	x	

PWV, pulse wave velocity; FeNO, fractional nitric oxide (NO) concentration in exhaled breath; T/N, technology and nutrition visit. Table 2 outlines the schedule we will follow during the study pertaining important benchmarks of enrollment, baseline visit (done pre-randomization), post visit (done after randomization), and closing out, or placing the participant off-study. The table is arranged according to the type of data collected (enrollment, interventions, assessments, instructive components). All follow-up components will be available to both intervention groups.

**Table 3:** Intervention Behavioral Component Content.

Week	Education Materials (Videos)	Social Cognitive Theory (Coaching)
1	Introduction to the FIREHOUSE study.	Goals for life.
2	Self-monitoring of diet and physical activity—making sense of the numbers.	Where am I? Establishing the relevance of behavior change.
3	Being a Calorie Detective. Portion control and empty calories.	Setting goals.
4	Introducing physical activity into your life. Finding time for fitness. Exercise safety.	Self-Reward. Turning goals into habits.
6	Being a Fat Detective. Healthy and unhealthy fats, the contribution of fat to total calorie intake.	Social support. Developing and working your social support network.
8	Building duration and intensity of aerobic exercise.	Problem solving: Barriers and setbacks. Introduction to the problem-solving model.
10	Changing seasons, special occasions, life events, and eating at restaurants.	Problem solving: Behavioral triggers and stimulus control.
12	The role of sleep and stress in weight gain and loss.	Problem solving: Stress management.
14	Adding color and fiber to your diet.	Problem solving: Emotional eating.
16	The role of breakfast and meal frequency in weight loss success.	Problem solving: Eliminating negative self-talk.
18	Snacking and sugar-sweetened beverages. Empty calories.	Problem solving: Food cravings, addictions, and habitual over-eating.
20	Building muscles with strength training.	Problem solving: Anticipating high-risk situations.
22	Weight loss plateaus.	Problem solving: Lapse and relapse.
24	Putting it all together; review of lifestyle recommendations.	Problem solving: Coping with lapses and setting new goals.

Table 3 provides a breakdown of the schedule and learning goals for the intervention group. This is organized by the week in the study, as well as the title of the lesson and the social cognitive theory aiming to be achieved. During the first month, the LoCalMed study group subjects receive weekly education and coaching remotely, which changes to bi-weekly in remaining months 2–6.

**Table 4:** Endpoints of the FIREHOUSE Trial.

	Outcome Measure	Description
**Primary Endpoint**	Body mass index (BMI) in kg/m^2^	Body mass divided by square of individual’s heights, with attempt to quantify and standardize amount of tissue mass across persons
**Secondary Endpoints**	FEV_1_	Usual spirometric technique with reproducibility and acceptability based on ATS/ERS guidelines. Allows best correlation with symptoms and pulmonary function
	Bioelectrical impedance analysis	InBody270 BIA scale. Measure lean body mass and total body fat percentage
	St. George’s Respiratory Questionnaire	Standardized/validated airways disease-specific survey to assess symptoms, activity hindrance, and overall impact
	Short Form 36	Standardized/validated general health survey to assess mental, emotional, and functional health status
	Pulse wave velocity	SphygmoCor (Atcor Medical) by carotid–femoral pulse discretion to measure vascular stiffness
	FeNO	NIOX VERO portable, to assess airway inflammation
	Vital Signs	BP, HR, RR, body temp, neck/waist/hip circumferences
	Electrocardiogram	Standard 12-lead ECG to assess axis changes
	Phlebotomy	Routine cell counts, metabolic panels, lipid panel. Possible further analysis of metabolomic fingerprints
**Exploratory Endpoints**	Microbiome	GenoTek Oragene-Gut personal stool collection kit
	Genome	DNA GenoTek Oragene-Discover saliva collection kit

FEV_1_, forced expiratory volume in one second; ATS/ERS, American Thoracic Society/European Respiratory Society; BIA, bio-electrical impedance analysis; BMI, body mass index; FeNO, fractionated exhaled nitrous oxide; ECG, electrocardiogram; BP, blood pressure; HR, heart rate; RR, respiratory rate. Table 4 details the primary, secondary, and exploratory endpoints of the study. Along with each endpoint is a description of what that measurement consists of.

**Table 5:** Interim Monitoring Bounds.

Interim Analysis	Completion of Subjects/Group	Critical Value Z ±	*p* Value
1	30	3.951	0.000
2	50	2.686	0.007
3	70	2.129	0.033

Interpolated boundaries with 3-look O’Brien Fleming stopping boundaries 140 subjects randomized with 2-sided α = 0.036, power = 80%. Table 5 describes the statistical measures which will be the basis for our interim analysis.

## Supplementary Materials

The following are available online at https://www.mdpi.com/1660-4601/17/18/6569/s1, Figure S1: Interim Monitoring Bounds. Interpolated stopping boundaries—z-score critical values for the 3-look design. Table S1: Study Procedures.

## Abbreviations

AE	Adverse Event/Adverse Experience
BIA	Bioelectric Impedance Analysis
BMI	Body Mass Index (kg/m^2^)
CFR	Code of Federal Regulations
COPD	Chronic Obstructive Pulmonary Disease
COVID-19	Coronavirus Disease-2019
CSOC	Clinical Study Oversight Committee
CTSI-CRC	Clinical and Translational Science Institute-Clinical Research Center
DHHS	Department of Health and Human Services
DSMB	Data and Safety Monitoring Board
ECG	Electrocardiogram
FDNY	Fire Department of New York
FeNO	Fractional Exhaled Nitric Oxide
FEV_1_	Forced Expiratory Volume in 1 second
FFR	Federal Financial Report
FFQ	Food Frequency Questionnaire
FIREHOUSE	Food Intake REstriction for Health OUtcome Support and Education
FVC	Forced Vital Capacity
FWA	Federal Wide Assurance
GCP	Good Clinical Practice
HIPAA	Health Insurance Portability and Accountability Act
HP	Health Program
ICF	Informed Consent Form
ICH	International Conference on Harmonization
IRB	Internal Review Board
LLN	Lower Limit of Normal
LoCalMed	Low Calorie Mediterranean Diet
MetSyn	Metabolic Syndrome
MOP	Manual of Procedures
MND	MyNetDiary
N	Number (typically refers to number of participants)
NIH	National Institutes of Health
NYU	New York University
OAD	Obstructive Airways Disease
OHRP	Office of Human Research Protections
OHSR	Office of Human Research Subjects
PI	Principal Investigator
PID	Participant ID Number
PPE	Personal Protective Equipment
PM	Particulate Matter
PWV	Pulse Wave Velocity
RCT	Randomized Clinical Trial
SARS-CoV-2	Severe Acute Respiratory Syndrome Coronavirus 2
SF-36	Short Form 36
SGRQ	St. George’s Respiratory Questionnaire
SOP	Standard Operating Procedure
US	United States
WTC	World Trade Center
9/11	11 September 2001
