# Supplementary material for: Food Intake REstriction for Health OUtcome Support and Education (FIREHOUSE) Protocol: A Randomized Clinical Trial
Source: Int J Environ Res Public Health. 2020 Sep 9;17(18):6569. doi: 10.3390/ijerph17186569 (PMC7559064; doi:10.3390/ijerph17186569)
Supplement: Supplementary file 1 [file ijerph-17-06569-s001.pdf]

International Journal of
Environmental Research and Public Health

Supplamentary Materail

Food Intake REstriction for Health OUtcome Support and Education (FIREHOUSE) Protocol: A Randomized Clinical Trial

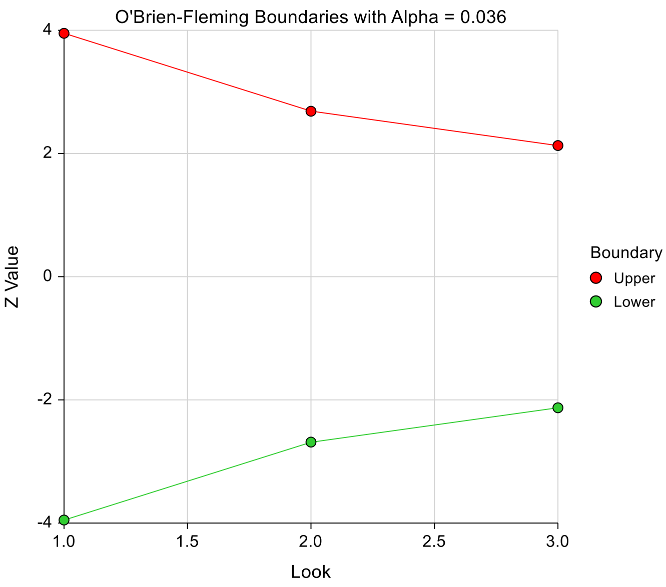

Figure 1. Interim Monitoring Bounds. Interpolated stopping boundaries—z-score critical values for the 3-look design.

Table S1. Study Procedures.

Table S1. Study Procedure (detail)						
**Activity**	**Telephone Screening**	**Screening**	**Baseline Visit**	**Randomization**	**Intervention**	**6 Month Visit**
**Study Team Procedures**						
Consent		X	X			
Medical History		X	X			
Physical Exam		X	X			X
Height/Weight		X	X			X
Vital signs		X	X			X
Fasting Visit (8-12 hrs)			X			X
Food Frequency Questionnaire		X				X
Study Intervention Information	X	X	X	X	X	X
Subject Survey		X	X			X
Deliver/Collect Questionnaires		X	X			X
Telephone Communication	X			X	X	
Electrocardiogram			X			X
Pulse Wave Velocity			X			X
Laboratory Assessments						
Chemistry/Lipid/Hepatic Panels			X			X
Genomics/Microbiomics			X			X
CBC with differential			X			X
Respiratory Assessments						
FE_{NO} testing			X			X
Spirometry			X			X

Abbreviations: CBC Complete Blood Count; **FE_{NO}** Fractional Exhaled Nitric Oxide;
